# JmjC Family of Histone Demethylases Form Nuclear Condensates

**DOI:** 10.3390/ijms23147664

**Published:** 2022-07-11

**Authors:** Marta Vicioso-Mantis, Samuel Aguirre, Marian A. Martínez-Balbás

**Affiliations:** Department of Molecular Genomics, Instituto de Biología Molecular de Barcelona (IBMB), Consejo Superior de Investigaciones Científicas (CSIC), 08028 Barcelona, Spain; mvmbmc@ibmb.csic.es (M.V.-M.); saibmc@ibmb.csic.es (S.A.)

**Keywords:** epigenetic regulation, histone demethylases (HDM), JmjC, IDR, phase separation

## Abstract

The Jumonji-C (JmjC) family of lysine demethylases (KDMs) (JMJC-KDMs) plays an essential role in controlling gene expression and chromatin structure. In most cases, their function has been attributed to the demethylase activity. However, accumulating evidence demonstrates that these proteins play roles distinct from histone demethylation. This raises the possibility that they might share domains that contribute to their functional outcome. Here, we show that the JMJC-KDMs contain low-complexity domains and intrinsically disordered regions (IDR), which in some cases reached 70% of the protein. Our data revealed that plant homeodomain finger protein (PHF2), KDM2A, and KDM4B cluster by phase separation. Moreover, our molecular analysis implies that PHF2 IDR contributes to transcription regulation. These data suggest that clustering via phase separation is a common feature that JMJC-KDMs utilize to facilitate their functional responses. Our study uncovers a novel potential function for the JMJC-KDM family that sheds light on the mechanisms to achieve the competent concentration of molecules in time and space within the cell nucleus.

## 1. Introduction

The regulated chromatin activity is the key event that allows multicellular organisms to support the process implicit in development, physiology, and homeostasis. It has become clear during the last decades that this control is mediated through histones and DNA modifications, the incorporation of histone variants, and the action of chromatin remodeling complexes [1]. One of the significant modifications, particularly relevant during development, is the histone lysine methylation, which encompasses the introduction of one (me1), two (me2), or three (me3) methyl groups to histones lysines or me1 and me2 to arginines [2]. Depending on their genomic localization and methylated residue, the different methylation states will imply transcriptional activation or repression [3]. Although histone methylation might impact chromatin structure, the central role of this modification is to be recognized by “reader” proteins that specify the downstream events. Early days, it was believed that histone methylation was irreversible. The discovery of the first histone lysine demethylase (KDM), KDM1A/LSD1, which removes methyl groups from histone H3 on lysine 4 (H3K4) [4], underscored the dynamic nature of histone methylation. Soon after that, the enzyme KDM2A/JHDM1A/FBXL11 was identified. It possesses a newly identified catalytic domain, the Jumonji-C (JmjC) domain [5]. KDM2A was the first member of an extended family of related demethylases, which coordinate iron to demethylate lysines using a-ketoglutarate (a-KG) as a cofactor, the JmjC domain-containing family of KDMs (from now on JMJC-KDM) [6,7]. Since these initial findings, our understanding of how these enzymes function has progressed rapidly. Numerous studies over the last decade have highlighted their role in many genomic processes such as transcription control [8,9], DNA replication [10,11], and DNA repair [12,13,14]. Notably, histone demethylase (HDM) activity alteration is linked to a wide spectrum of human diseases, including mental disorders and cancer [15,16]. In this way, KDMs have become new attractive therapeutic targets [17,18].

The members of the JMJC-KDM family play an essential role in gene expression regulation, chromatin organization, and cellular homeostasis [19]. In most cases, this was attributed to their demethylase activity. However, accumulating evidence demonstrates that these proteins play roles distinct from histone demethylation [20,21]. These data lead us to hypothesize that the major regulatory function of these proteins might rely not only on their catalytic capacity but other domains could be involved. Thus, whether JMJC-KDMs have any common domain that modulates their activity arises.

Recently, it has been shown that multiple components of the transcriptional machinery cluster via phase separation to efficiently accomplish their function during transcription [22,23,24]. These transcriptional hubs or condensates include RNA polymerase II (RNAPII) [22], cofactors (Mediator, Bromodomain-containing 4 (Brd4)) [22,25], transcription factors (Oct4, Smad) [26,27], and even histone modifications themselves are involved in the process [28,29,30]. Phase separation is a dynamic process that allows the formation of biomolecular condensates by establishing weak, multivalent interactions. These commonly use electrostatic and hydrophobic residues [31,32]. These interactions are often driven by intrinsically disordered regions (IDR) and low-complexity domains [33,34]. First analyses of the JMJC-KDMs indicated that a large part of the amino acids of these proteins is not organized into known domains. A closer analysis clarified that they belong to disordered regions. Thus, the possibility exists that JMJC-KDMs utilize these domains to cluster into biomolecular condensates to facilitate their function. 

Here, we show that all JMJC-KDMs contain intrinsically disordered regions, which in some cases reach up to 70% of the protein. Our data revealed that plant homeodomain finger protein 2 (PHF2), KDM2A, and KDM4B could phase separate. Finally, our PHF2 molecular analysis suggests that clustering is a common feature that JMJC-KDMs might utilize in addition to their catalytic activity to promote their functional responses. Thus, our study uncovers a novel molecular property of the JMJC-KDM family that sheds light on the ways to achieve the competent concentration of molecules inside the cells. 

## 2. Results

### 2.1. JMJC-KDMs Are Intrinsically Disordered Proteins

**Figure 1:**
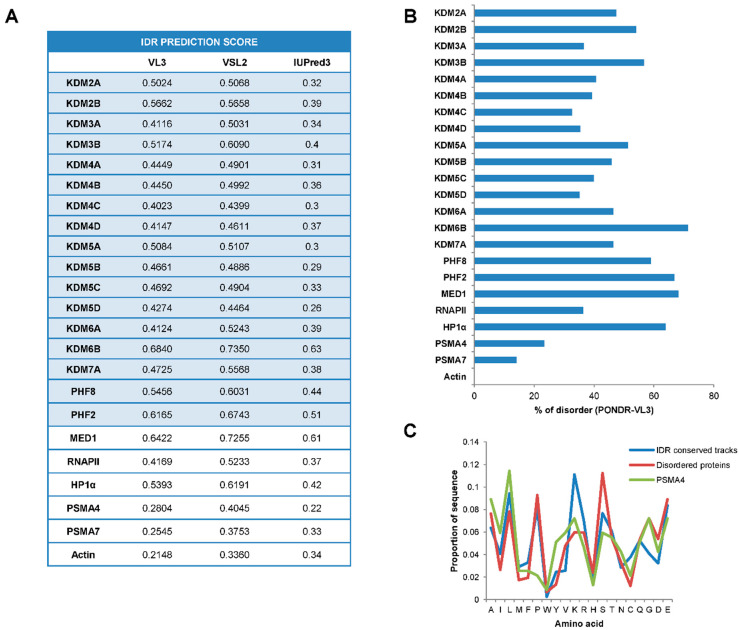
JMJC-KDMs are intrinsically disordered proteins. (**A**) Table representing the IDR prediction score of JMJC-KDMs using PONDR-VL3, VSL2, and IUPred3 algorithms. (**B**) Disorder percentage prediction of human JMJC-KDMs using PONDR-VSL3 algorithm (length of disordered segments > 50 amino acids). (**C**) Amino acid composition of the IDR conserved tracks between KDM families, and disordered proteins. The ordered protein PSMA4 was also included as a negative control (see Methods).

In the last years, numerous works have shed light on the impact of IDRs on transcriptional regulation [35,36]. Since a large part of JMJC-KDMs does not encompass any identified structure, we questioned whether JMJC-KDMs belong to the group of intrinsically disordered proteins. To assess this, we analyzed the sequence of the JMJC-KDMs searching for disordered regions using the following algorithms: PONDR-VL3, IUPred, and VSL2 (see Material and Methods). Overall, the three algorithms agreed on the significant disorder score of these proteins (Figure 1A and Figure S1). In particular, PONDR-VL3 revealed a disorder score ranging from 0.68 (KDM6B) to 0.40 (KDM4), considerably higher than the values obtained when analyzing the proteasome components PMS4A and PMSA7, and actin, well-known structured proteins used as negative controls (Figure 1A and Figure S1). In addition, a compelling amino acids proportion (from 71.39% KDM6B to 32.67% KDM4C using PONDR-VL3) exists in disordered domains (Figure 1B). These values are higher than the obtained for the negative controls proteasome subunit alpha type-4 (PSMA4) (23.37%) and similar to examples of disordered proteins such as Mediator 1 (MED1) (68.25%), RNAPII (36.35%), or heterochromatin protein 1 (HP1)α (63.87%) [25] (Figure 1B). 

Interestingly, 99% of identified IDRs on JMJC-KDMs contain amino acid tracks conserved within the family (see Material and Methods). These tracks were enriched in lysines (K) (9.7%) and prolines (P) (9.1%) as well as in hydrophobic amino acids (leucines, L, 10.3%) (Figure 1C), features that have been associated with the ability to phase separate [33].

### 2.2. JMJC -KDMs Are Prone to Phase Separate

**Figure 2:**
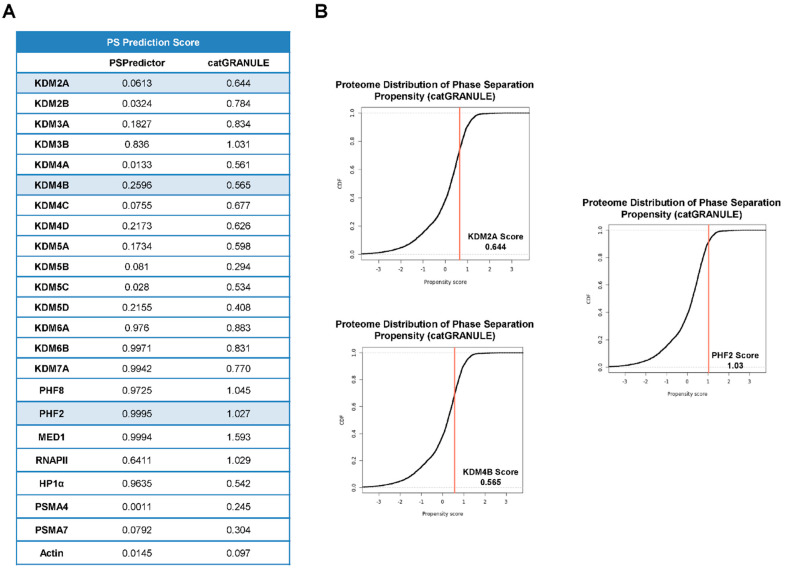
JMJC-KDMs are prone to phase separate. (**A**) Predictions of JMJC-KDMs putative capacity to phase separate, determined with the PSPredictor (on the left) and the catGRANULE (on the right) algorithms. (**B**) Distribution of phase separation propensity of KDM2A, KDM4B, and PHF2 determined by the catGRANULE algorithm.

IDRs drive condensate formation via phase separation, so we used the PSPredictor and catGRANULE algorithms (see Material and Methods) to predict JMJC-KDM’s phase separation propensity. Both tools returned high scores (from 0.99 PHF2 to 0.01 KDM4A for PSPredictor) (Figure 2A), in some cases similar to proteins known to be involved in phase separation (e.g., MED1, 0.99, RNAPII, 0.64, or HP1α, 0.96) and consistently higher than the proteasome proteins PSMA4 (0.001), PSMA7 (0.079) or actin (0.014) (Figure 2A). Altogether these data point to JMJC-KDMs as disordered proteins with the potential to undergo phase separation. 

**Figure 3:**
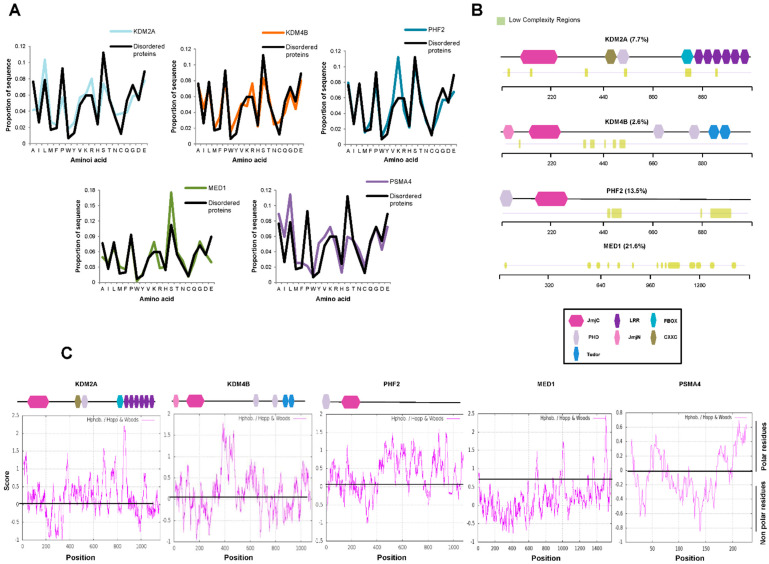
JMJC-KDMs contain low-complexity domains. (**A**) Amino acid composition of KDM2A, KDM4B, PHF2, MED1, PMSA4, and disordered proteins defined by the presence of 50 residues fragment whose IUPRED median score is at least 0.55 and that it is not found in Pfam. (**B**) Analysis of the presence of low-complexity domains in KDM2A, KDM4B, PHF2, and MED1 using the SEG algorithm. The percentage of low-complexity regions is indicated. Low-complexity regions are depicted in green. A schematic representation of the KDM domains is shown on the bottom. (**C**) The hydrophobicity profile of KDM2A, KDM4B, PHF2, MED1, and PMSA4 was determined using the ExPASy website with the Hopp and Woods scale and a sliding window of 21. A scheme of the KDMs domains is depicted on top of each panel.

To further analyze the ability of JMJC-KDMS to cluster together via phase separation, we chose three KDMs among those with high (PHF2), low (KDM4B), and medium (KDM2A) scores according to both predictors and that belong to different families (Figure 2A,B). Our analysis did not include KDM6 family members that show very high scores because it has recently been demonstrated that their two members undergo phase separation [9,37]. The amino acid composition has also been shown to play an essential role in the IDR-mediated formation of transcriptional condensates. We thus analyzed the amino acid composition of the selected KDMs and found an enrichment of lysines and serines for PHF2; glutamate, serine, and lysine for KDM4B; glutamate, arginine, and leucine for KDM2A (Figure 3A) compared to the average in the mouse disordered proteins [38,39]. These residues are associated with the ability to phase separate [33]. We used MED1 and PMSA4 proteins as positive and negative controls, respectively.

Moreover, disordered regions frequently concur with low-complexity domains that contribute to phase separation [25,31,33,40]. Then, we check whether the KDMs are enriched in low-complexity domains using the SEG algorithm (see Material and Methods). As shown in Figure 3B, between 2.6% (KDM4B) and 13.5% (PHF2) of KDM sequences were predicted to contain low-complexity segments. Furthermore, it has been previously described that hydrophobic interactions might be crucial for biomolecular condensate formation [23,40]. Then, we analyze the hydrophobicity of the selected JMJC-KDMs, finding the presence of hydrophobic tracks (Figure 3C). Thus, our data suggest that the amino acid composition of JMJC-KDMs primes them to be involved in the phase separation process. 

### 2.3. KDM2A, KDM4B, and PHF2 KDMS Cluster In Vitro and Inside the Cell

**Figure 4:**
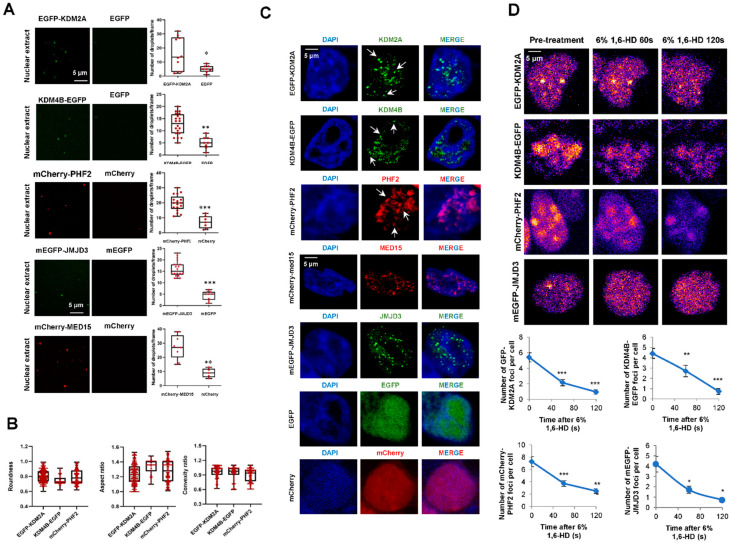
JMJC-KDMs undergo LLPS in vitro and inside the cell. (**A**) EGFP-KDM2A, KDM4B-EGFP, mCherry-PHF2, EGFP-JMJD3, mCherry-MED15, EGFP, and mCherry proteins were analyzed using droplet-formation assays in nuclear extracts at room temperature in the presence of 150 mM NaCl. Quantification of the droplets is displayed on the right. Data are the mean ± SEM. * *p* < 0.05, ** *p* < 0.01 and *** *p* < 0.001 (Student’s *t*-test). Droplets in 5 fields from three biologically independent experiments were quantified. Scale bar, 5 μm. (**B**) Boxplots represent the roundness, convexity, and aspect ratio features of the fluorescent-tagged KDMs droplets. Data are the mean ± SEM. Quantifications correspond to droplets in 5 fields from three biologically independent experiments for each protein. (**C**) Confocal microscopy images of HEK293T cells transfected with EGFP-KDM2A, KDM4B-EGFP, mCherry-PHF2, EGFP-JMJD3, mCherry-MED15, EGFP, or mCherry and visualized with the fluorescence tag and DAPI to stain the DNA. Images are representative of 3 biologically independent experiments. Scale bar, 5 μm. (**D**) HEK293T cells were transfected with 0.05 ug plasmid encoding EGFP-KDM2A, KDM4B-EGFP, mCherry-PHF2, or EGFP-JMJD3; they were treated with 6% 1,6-Hexanediol for 5 min and imaged at 60 and 120 s. Nuclei were visualized with DAPI (blue). Quantification of the nuclear puncta displayed per cell along the treatment is shown on the right. Data are the mean ± SEM. * *p* < 0.05, ** *p* < 0.01; *** *p* < 0.001 (Student’s *t*-test). *n* = 30 transfected cells were quantified; Images are representative of three biologically independent experiments. Scale bar, 5 μm.

Considering the potential of the analyzed KDMs to phase separate and their role in transcriptional control, we further proceeded to study the capacity of these proteins to form biomolecular condensates. We fused KDM2A and KDM4B to EGFP, and PHF2 to mCherry fluorescence tags (Figure S2A), we expressed them in HEK293T cells (Figure S2B), and we performed in vitro droplet assays using nuclear extracts. Our data show that the selected KDMs formed droplets that do not appear when we overexpressed EGFP or mCherry alone, used as negative controls, suggesting that the droplets can be attributed to KDM proteins and not to the fluorescence tags (Figure 4A). The number of droplets was similar to those obtained for MED15 and Jumonji domain-containing protein 3 (JMJD3), two proteins that experience liquid-liquid phase separation (LLPS) [9,41], used as positive controls. Furthermore, the obtained droplets showed features [roundness, convexity, and aspect ratio] characteristic of a liquid-like nature (Figure 4B).

Next, we analyzed the ability of fluorescence KDMs to form puncta in fixed cells, observing that when these KDMs were overexpressed in HEK293T cells, condensates could be detected as nuclear puncta (Figure 4C). It has been previously demonstrated that the aliphatic alcohol 1,6-hexanediol disrupts the hydrophobic interactions that maintain the phase-separated droplets. Thus, we tested the sensitivity of the KDM’s condensates to 1,6-hexanediol. The data demonstrated that the treatment reduced the number and size of KDMs puncta (Figure 4D) without affecting the total amount of protein (Figure S2C). We also analyzed the formation of puncta by the endogenous PHF2. Immunofluorescence experiments in HEK293T and NIH3T3 cells showed that the endogenous protein could form nuclear condensates (Figure S2D), ruling out that the observed puncta were due to an overexpression artifact.

These data suggest that JMJC-KDMs condensate and that these condensates are a separated phase inside the cell.

### 2.4. KDM2A, KDM4B, and PHF2 Condensates Correlate with Transcription 

**Figure 5:**
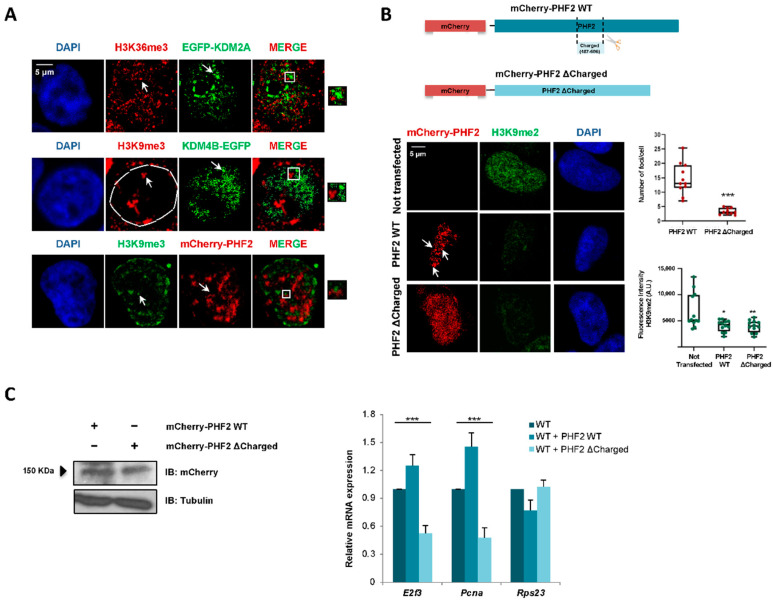
KDM2A, KDM4B, and PHF2 condensates correlate with transcription. (**A**) HEK293T cells were transfected with 0.05 μg of EGFP-KDM2A, KDM4B-EGFP, and mCherry-PHF2, and the localization of the active (H3K36me3) or the repressive (H3K9me3) histone marks were analyzed using immunofluorescence staining with anti-H3K36me3 or anti-H3K9me3. The JMJC-KDMs localization was determined following the fluorescence signal. Nuclei were visualized with DAPI (blue). Scale bar, 5 μm. The images are representative of three independent experiments with similar results. (**B**) mCherry-PHF2 WT and mCherry-PHF2 ΔCharged expression vectors (top panel) were transfected into HEK293T cells. The formation of nuclear puncta was analyzed by following the fluorescence signal. The levels of H3K9me2 were determined by immunofluorescence using anti-H3K9me2 antibody. The boxplots on the right show the number of foci per cell and the H3K9me2 fluorescence intensity. *n* = 30 transfected cells for condition. Data show the mean ± SEM. For the number of foci per cell, statistic comparisons were made between cells overexpressing PHF2 WT and cells overexpressing PHF2 ΔCharged (*** *p* < 0.001, Student’s *t*-test). For fluorescence intensity, comparisons were performed between not transfected cells and cells overexpressing PHF2 WT (* *p* < 0.05, Student’s *t*-test) and between not transfected cells and cells overexpressing PHF2 ΔCharged (** *p* < 0.01, Student’s *t*-test). Images are representative of 3 biologically independent experiments. Scale bar, 5 μm. (**C**) PHF2 WT and PHF2 ΔCharged were transfected. 24 h later, total protein extracts were prepared, and the expression levels of PHF2 WT and the mutant were determined by immunoblot using anti-mCherry antibody. The image shown is representative of two independent experiments (left). The expression levels of *E2f3* and *Pcna* genes were quantified by qPCR in NIH3T3 cells (WT, dark blue) expressing PHF2 WT (WT + PHF2 WT, blue) or PHF2 ΔCharged (WT + PHF2 ΔCharged, light blue) (right). As a negative control, the mRNA levels of the housekeeping gene *Rps23* were measured. Error bars represent SEM. Statistic comparisons were made between the three groups by means of one-way ANOVA tests (*** *p* < 0.001). Results are representative of three biologically independent experiments.

The data above indicate that JMJC-KDMs undergo LLPS in the nucleus; then, we hypothesized that clustering propensity could contribute to the transcriptional regulation mediated by JMJC-KDMs. To test this idea, we analyzed the localization of KDM condensates with respect to the repressive H3K9me3 and active H3K36me3 histone marks by immunofluorescence assays. These histone marks were used because they form nuclear foci and are landmarks of transcriptional repression and activation, respectively. KDM4B-EGFP and mCherry-PHF2 nuclear condensates excluded the repressive mark H3K9me3 (Figure 5A), consistent with their role in transcription activation. Moreover, according to its role as a transcriptional repressor, EGF-KDM2A puncta were excluded from condensates marked by the active mark H3K36me3 (Figure 5A).

To further analyze the contribution of the IDRs to transcription regulation, we selected the PHF2 protein (which has been previously studied by our lab) to construct a mutant protein that lacks part of its IDR. In particular, we depleted a strikingly lysine-enriched region (amino acids 487-806) (Figure S3A) that is widely conserved among mammals (Figure S3B). We named this mutant PHF2 ΔCharged (see Material and Methods). Next, we tested its ability to form nuclear puncta. When compared to the PHF2 WT protein, the mutant could not phase separate (Figure 5B). Thus, we concluded that the lack of the charged region impacted the phase separation potential of PHF2. Interestingly, the mutation did not affect the ability to demethylate H3K9me2 (Figure 5B). Next, we decided to study whether the lack of phase separation of the PHF2 mutant translated into a functional impairment, affecting the transcription regulation. We measured the expression levels of some PHF2-dependent genes (*E2f3* and *Pcna*, [42]) in NIH3T3 cells that overexpress either PHF2 WT or PHF2 ΔCharged (Figure 5C, left). While the WT protein increased the mRNA level of these genes, the mutant acted as a dominant-negative protein hindering their expression (Figure 5C, right). The housekeeping gene *Rps23*, which was used as a negative control, was not affected by the deletion of the charged track (Figure 5C). Thus, the capacity of PHF2 to activate transcription seems to be related to the presence of the charged domain in its IDR.

Altogether these results support a model in which JMJC-KDM might use a common mechanism that might regulate their outcome by forming IDR-driven condensates. Thus, our study uncovers a novel molecular property of the JMJC-KDM family that sheds light on the ways to achieve the competent concentration of molecules inside the cells. 

## 3. Discussion

The requirement of different molecules to perform a function in the correct concentration, time, and space inside the cells is a central question. Cells solve this problem continuously in a dynamic way. With the evolution, some organelles have developed the formation of a membrane to facilitate biological functions such as mitochondria, nuclei, Golgi apparatus, etc. Other exists in a membraneless fashion (Cajal bodies, nucleoli, or signaling bodies). Recently, the membraneless condensates have received much attention; however, how they are formed and their function is unclear. In recent years, it has been shown that many of these condensates have liquid-like properties and are formed by phase separation [31]. Phase separation plays a myriad of biological functions, from signal transduction, filament polymerization, and chromatin structure to transcriptional control [43]. Moreover, phase separation is related to many diseases, from cancer to neurodegenerative disorders [44,45,46]. 

Recently, the contribution of phase separation to transcription regulation has become an intense area of research [38]; however, the molecular mechanisms through which this control is exerted are not fully understood. Our study reveals a common feature of the chromatin-acting enzymes, the JMJC-KDMs. We show that they are prone to phase separation and that representative examples from nuclear condensates are important for their functional outcome. 

Bearing this in mind, we can question the role of the JMJC-KDMs condensates. We will discuss some relevant aspects that could be affected by the phase separation of these regulatory proteins, such as the chromatin structure and the machinery involved in transcription.

### 3.1. JMJC-KMD Condensates May Affect Their Functional Substrate, the Chromatin Structure

Although our results for PHF2 suggest that phase separation might not affect its catalytic activity, an interesting possibility is that phase separation might affect the catalytic activity of some JMJC-KDM enzymes, as recently demonstrated for the JMJC-KDM KDM6A [37]. That, in turn, might have a profound effect on chromatin structure and/or phase separation itself. Indeed, it has been described that histone modifications can affect chromatin phase separation. For instance, in the presence of BRD4 protein, highly acetylated chromatin forms a new phase-separated state similar to the chromatin subcompartments [28]. The droplets formed by BRD4 have a repelling effect on chromatin, which leads to the remodeling of chromatin structure [47]. These data suggest that changes in KDM’s activity mediated by phase separation might affect chromatin structure.

On the other hand, it is well known that the chromatin structure also affects the phase separation process [28,48]. Phase separation droplets tend to be formed in the chromatin structure with open and low-density areas [49]. The surrounding chromatin has a certain mechanical resistance to the formation of droplets [50,51]. Then, changes in the methylation status might impact chromatin conformation and its functional outcome.

### 3.2. JMJC-KMD Condensates May Affect Transcriptional Machinery Function

RNAPII is a key molecule in eukaryotic gene transcription. It forms phase-separated condensates through the interaction of its C-terminal domain (CTD) with many components of the transcriptional machinery [52]. In particular, Mediator and Brd4 factors and coactivators stabilize the pre-initiation complex at gene promoters [53]. Other key components of the transcriptional machinery, the transcription factors, have also been shown to contribute to transcription through a phase separation process [26,27]. The current model proposes that phase-separated condensates regulate gene transcription [23,54,55]. At promoter, an essential condensate formed by Mediator, transcription factors, coactivators, and non-phosphorylated RNAPII allows initiation. Downstream of the initiation site, phosphorylated RNAPII, nascent RNA, elongation factors, RNA processing factors, and specific elongation coactivator form a condensate that allows elongation [22,56]. In addition to the promoter, it has been demonstrated that enhancers and enhancer clusters participate in gene regulation through phase separation [54]. JMJC-KDMs are essential cofactors for transcription. They contribute to transcriptional control at promoter and enhancer sites and during elongation. Thus, KDM’s ability to drive or form part of nuclear condensates might be critical to transcriptional hub formation. KDM’s condensates might concentrate transcription factors, cofactors, and the general elongation factors at promoters or enhancers in a compartment to make transcription kinetically more efficient, as proposed for other factors [22,26,55]. If this was the case, JMJC-KDM biomolecular condensates would work as transcriptional hubs that compartmentalize coactivators and transcriptional machinery to favor the transcription process, as recently proposed [9,37].

In addition, other possible scenarios can be envisioned.

- JMJC-KDM condensates may physically insulate the transcriptional machinery from its regulators to prevent inactivation. In this way, KDM’s condensates maintain the transcriptional process ongoing;
- They could stabilize complexes formed by multiple components, which generally are involved in transcription initiation and elongation;
- Condensate formation might facilitate contacts between distal genomic regions that would favor transcription burst frequency or intensity, as has been suggested for KDM6A and KDM6B [9,37];
- JMJC-KDMs condensation might serve as a mechanism for accessing the compacted and closed chromatin, as demonstrated for Kruppel-like factor 4 (KLF4) during reprogramming [55]; the deformable properties of many IDRs might facilitate the access of these enzymes into the compacted facultative or constitutive heterochromatin to favor nucleosome unwrapping. This may be particularly important during development or reprogramming, where LLPS and KDMs have been shown to play a relevant role.

Phase separation is linked to the occurrence of many pathologies, such as cancer, neurodegeneration, or infections [44,45,46]. Recently, the histone demethylase KDM6A tumor suppressor capacity has been shown to be dependent on the ability of its IDR to phase separate [37]. Interestingly, many mutations on JMJC-KDMs that lead to diseases occur in their IDRs. In particular, PHF2 nonsense mutations in exons 12, 16, and 18 have been associated with different cancer types [57]. In all cases, PHF2 IDR has been compromised. A more detailed understanding is required of the direct or indirect involvement of the IDR from JMJC-KDM on pathologies, of the possible condensing proteins mechanism in this process, and of their potential as therapeutic targets. 

Our study uncovers a novel molecular property of the JMJC-KDM family that sheds light on the ways to achieve the competent concentration of molecules inside the cells. Thus, JMJC-KDM’s condensate formation might be a general and key physical platform for the regulation of chromatin structure and activity. 

## 4. Materials and Methods

### 4.1. Sequence Analysis and Predictions

To calculate the protein disorder estimations, three prediction algorithms were used, PONDR-VL3 [58], IUPred [59], and PONDR-VSL2 [60]. The predictors provide a score between 0 and 1 for each amino acid that was above 0.5 and lay within a disordered region longer than 50 AA. The phase separation property of each protein was determined using the PSPredictor and catGRANULE [61] predictors. Low-complexity domains were determined using the SEG algorithm with the MobiDB database [62]. The Prot Pi Protein Tool website was used to analyze amino acid composition. The presence of 50 residues fragments whose IUPRED median score was at least 0.55 and that was not found in Pfam was used as criteria to define disorder proteins so that functional domains were avoided. The hydrophobicity was estimated with the ExPASy website [63] using the Hopp and Woods scale [64] and a sliding window of 21. The conservation of PHF2 IDR region was analized using Multiz alignments [65].

### 4.2. Amino Acid Composition of Conserved IDRs within KDM Families

The IDR conservation between the members of each JMJC-KDM family was assessed using the Basic Local Alignment Search Tool (BLAST ^®^) [66]. Then, to calculate the amino acid composition of these conserved IDRs, the number of each specific amino acid in the conserved sequences was divided by the total number of amino acids in the conserved sequences. This way, the amino acid proportion within a KDM family was calculated for each amino acid (e.g., proportion of K in KDM4 conserved tracks = number of K in KDM4 conserved tracks/number of total amino acids in KDM4 conserved tracks). Finally, the total amino acid composition of the conserved IDRs was calculated as the mean between the amino acid proportions for the conserved IDRs in each family (e.g., total K content in IDR conserved tracks = mean of the proportion of K in KDM2 conserved tracks, KDM3 conserved tracks, KDM4 conserved tracks, KDM5 conserved tracks, KDM6 conserved tracks, and KDM7 conserved tracks).

### 4.3. Cell Culture and Cell Treatments 

Human HEK293T and mouse NIH3T3 cells were grown in DMEM supplemented with 10% of fetal bovine serum (Gibco) and 1% of Penicillin/Streptomycin [67]. 

### 4.4. Plasmids 

pCDNA-FLAG-EGFP-KDM2A and pCDNA-KDM4B-EGFP were kindly provided by Dr. Till Bartke (IFE Helmholtz Munich, Neuherberg, Germany) and Dr Thomas Jenuwein (Max Planck Institute of Immunobiology and Epigenetics, Freiburg, Germany), respectively. To construct mCherry-PHF2, PHF2 was extracted from the p3xFLAG-PHF2 vector (kindly provided by Dr. Jiemin Wong, East China Normal University, Shanghai, China) through digestion with BamHI restriction enzyme and inserted into BamHI digested pCMV-mCherry-C1 plasmid. p3xFLAG-PHF2 ΔCharged vector was obtained from p3xFLAG-PHF2 through PCR amplification with specific primer pairs (Table S1) so that the deleted region was excluded. mCherry-PHF2 ΔCharged plasmid was obtained by replacing the PHF2 from the mCherry-PHF2 vector with the ΔCharged mutant version, which was extracted from the p3xFLAG-PHF2 ΔCharged digesting with BamHI. pCMV-mEGFP-JMJD3 was cloned as described in [9]. The mCherry-MED15 plasmid was kindly provided by Dr. R. Young (Massachusetts Institute of Technology, Cambridge, MA, USA).

### 4.5. Antibodies 

Antibodies used were anti: DAPI (Fisher Scientific, Madrid, Spain, D1306), β-TUBULIN (Merck Millipore, Darmstadt, Germany, MAB3408), H3K9me3 (Abcam, Cambridge, UK, ab8898), H3K9me2 (Abcam, Cambridge, UK 1220), H3K36me3 (Abcam, Cambridge, UK ab9050), PHF2 (Cell Signaling, Danvers, MA, USA, D45A2), mCherry (Fisher Scientific, Madrid, Spain, MA5-32977), and GFP (Roche, Basil, Switzerland, 11814460001). 

### 4.6. RNA Extraction and qPCR

Total RNA was prepared using TRIZOL reagent (Invitrogen, Waltham, MA, USA), following the manufacturer’s instructions. A total of 200-1000 ng of RNA was used for reverse transcription using a High Capacity cDNA Reverse Transcription Kit (Invitrogen, Waltham, MA, USA). qPCR was performed with SYBR Green (Roche, Basil, Switzerland) in a QuantStudio™ 5 Real-Time PCR System (Fisher Scientific, Madrid, Spain, A34322) with specific primer pairs (Table S1).

### 4.7. Western Blot

Immunoblotting was performed following the standard method. An ECL kit (Merck, Darmstadt, Germany, Amersham GE10600002) was used to visualize the results.

### 4.8. Droplet Assays in Nuclear Extracts

20 × 10 ^6^ HEK293T cells were transfected using 5 μg of the vector encoding KDMs cDNA fused to mEGFP or mCherry as previously described [68]. Nuclear extracts were prepared at a 4 mg/mL concentration. They were used for a droplet-formation assay by 1:1 diluting them with droplet buffer (10% glycerol, 20 mM HEPES). The final droplet buffer conditions were 20 mM HEPES, 150 mM NaCl, 15% glycerol, 3.75 mM EGTA, 2.5 mM MgCl_2_, 1.25 mM CaCl2. The reactions were incubated for 30 min and loaded onto a glass-bottom 384-well plate (Cellvis P384-1.5H-N) for 5 min. Then, they were imaged on an Automated Inverted Microscope Leica Thunder 3D Live Cell using a 63× water immersion objective (NA = 1.2).

### 4.9. Droplets Liquid-like Features Quantification

A quantification of the shape descriptors “aspect ratio” and “roundness” of droplets was performed with the Fiji plugin “Analyze particles”. The Fiji macro “Calculate Convexity and Solidarity” was used to quantify droplet “convexity” [32,69]. Each droplet was individualized as an object by fixing the threshold. The results shown in the graph correspond to n = 75 droplets.

### 4.10. 1,6-Hexanediol Treatment for Live Imaging Cells

20 × 10^6^ HEK293T cells growing on glass dishes coated with 5 μg/mL of poly-D-lysine were transfected with 0.05 μg of EGFP-KDMs and mCherry-PHF2 vectors. They were imaged on a 37 °C heated stage of a Zeiss LSM780 Confocal/Multiphoton using Zen software to determine a baseline using the X detector and a 40x water objective. After the fifth acquisition, 1,6-Hexanediol (Merck, Darmstadt, Germany, #240117) was added to the culture medium at a final concentration of 6%, and images were again taken for 5 min. Raw images were analyzed using Fiji software for subsequent quantifications. Representative images of puncta disassembly at 60 and 120 s are presented.

### 4.11. Indirect Immunofluorescence 

Immunofluorescence assays were performed as previously described [70]. Basically, cells were fixed with 4% paraformaldehyde (20 min) and permeabilized with PBS-Triton X-100 (0.5%). They were blocked in 0.5% BSA (in PBS with 0.1% Triton X-100) for 1 h at room temperature. Thus, they were incubated overnight at 4 °C with primary antibodies. Next, cells were incubated with Alexa-conjugated secondary IgG antibodies (Jackson ImmunoResearch, Cambridge, UK). DAPI (Merck, Darmstadt, Germany) was added for 2 hat room temperature. The intrinsic fluorescence of EGFP and mCherry was imaged without using primary/secondary antibodies. Images were obtained using a Leica SP5 confocal microscope utilizing LAS-AF software. 

### 4.12. Focus Calling (Immunofluorescence, 1,6-Hexanediol Treatment)

Foci were called using the “Object Counter 3D” plugin in Fiji. For each image, the “threshold” was set in the way that each focus could be seen as an individual object. 

### 4.13. Statistical Analysis

Quantitative data were expressed as mean and standard error mean (SEM) (for immunofluorescence counting and RNA transcription experiments). At least two to three biologically independent experiments were carried out for each experiment. The significance of differences was determined using Student’s *t*-test for two group comparisons, and one-way ANOVA tests for multiple groups comparisons (* *p* < 0.05; ** *p* < 0.01, *** *p* < 0.001).

## Data Availability

All relevant data supporting the key finding of this work are available in Supplementary Information and within the article.

## Supplementary Materials

The following supporting information can be downloaded at: www.mdpi.com/article/10.3390/ijms23147664/s1.
