# Supplementary material for: JmjC Family of Histone Demethylases Form Nuclear Condensates
Source: Int J Mol Sci. 2022 Jul 11;23(14):7664. doi: 10.3390/ijms23147664 (PMC9319511; doi:10.3390/ijms23147664)
Supplement: Supplementary file 1 [file ijms-23-07664-s001.zip › ijms-1692828-supplementary.pdf]

SUPPLEMENTARY INFORMATION

This file contains the Supplementary Figures, Supplementary Tables and Supplementary Figure Legends corresponding to the manuscript: "**JmjC family of histone demethylases form nuclear condensates** "

Marta Vicioso-Mantis, Samuel Aguirre, and Marian A. Martínez-Balbás

SUPPLEMENTARY FIGURE LEGENDS

Figure S1. JMJC-KDMs contain large intrinsically disorder regions

Disorder prediction of human JMJC-KDM proteins using PONDR-VL3 algorithm (Peng et al., 2005). The Residue number in the protein and the score returned by the PONDR algorithm are displayed on the x and y-axes respectively.

Figure S2. KDM2A, KDM4B and PHF2 form nuclear condensates

(A-B) EGFP-KDM2A, KDM4B-EGFP and mCherry-PHF2 expression vectors (A) were transfected into HEK293T cells. 24 h later total protein extracts were prepared and the expression levels of KDM2A, KDM4B and PHF2 were determined by immunoblot using anti GFP (KDM2A and KDM4B) and anti PHF2 (B). The image shown is representative of two independent experiments.

(C) EGFP-KDM2A, KDM4B-EGFP and mCherry-PHF2 plasmids were transfected into

HEK293T cells and treated or not with 6% 1,6-HD. Total protein extracts were prepared

and the expression levels of KDM2A, KDM4B and PHF2 were determined by immunoblot using the indicated antibodies. The image shown is representative of two independent experiments.

(D) HEK293T and NIH3T3 cells were fixed and endogenous PHF2 was visualized by immunostaining assay. Confocal microscopy images display puncta formed by endogenous PHF2. The images are representative of three biologically independent experiments. Scale bar, 5 μm.

Figure S3. PHF2 IDR is rich in charged amino acids.

(A) Schematic representation of the amino acid composition of PHF2 IDR. The percentages of lysines (purple), serines (pink), as well as basic, acid and charged amino acids are indicated at the bottom part of the panel. The grey-highlighted track corresponds to the lysine-enriched region (amino acids 487-806) deleted in PHF2 ΔCharged.

(B) UCSC tracks displaying the conservation of PHF2 IDR charged region (541-509 AA) among vertebrate species using Multiz alignments (Blanchette et al., 2004).

Supplementary Table S1. List of primers used in this study.

Region		Forward primer (FW)	Reverse primer (RV)
cDNA	*E2f3* mRNA	GGCCCATTGAGGTTTACTTG	ACCGAGCAGTCACTATGTC
	Pcna mRNA	GGGTGAAGTTTTCTGCAAG	GCAAACGTTAGGTGAACAGG
	Rps23 mRNA	CGTCAGGGTGCAGCTCATTA	GGCACGAACGCTGTGATCTT
Cloning for overexpression	*Phf2* (for ΔCharged mutant)	GAGAAGGAAGAACCTGACTCGTTACTGAAGAT	TAACGAGTCAGGTTCTTCCTTCTCCCGGTCTC

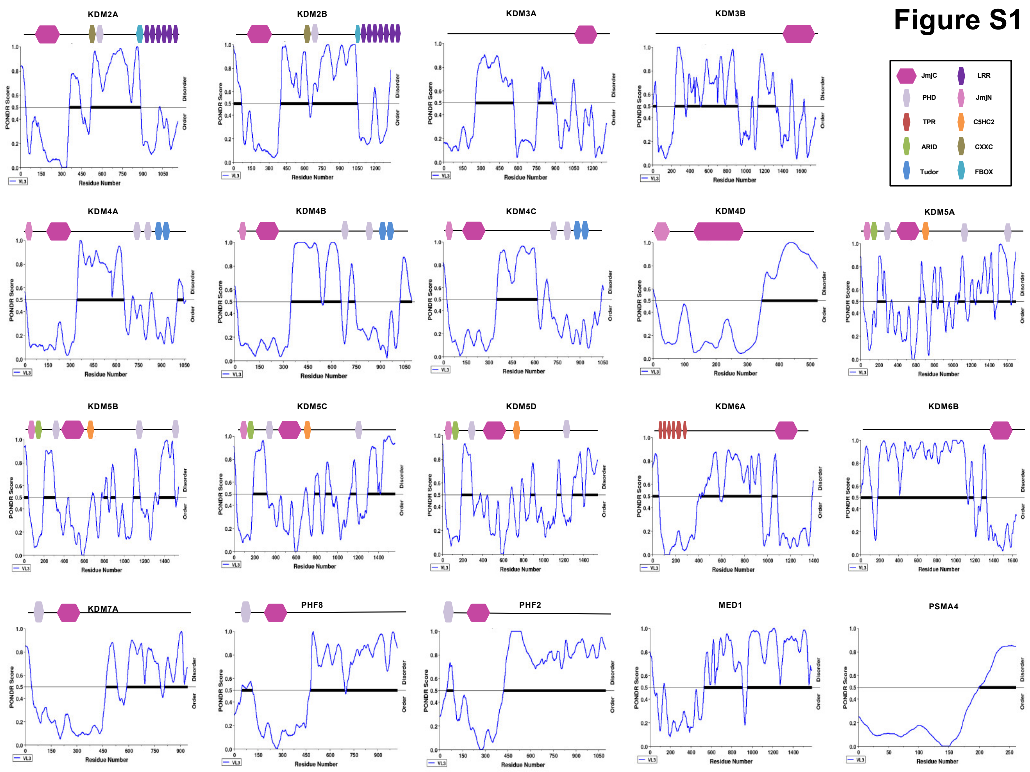

Figure S1. JMJC-KDMs contain large intrinsically disorder regions. Disorder prediction of human JMJC-KDM proteins using PONDR-VL3 algorithm (Peng et al., 2005). The Residue number in the protein and the score returned by the PONDR algorithm are displayed on the x and y-axes respectively.

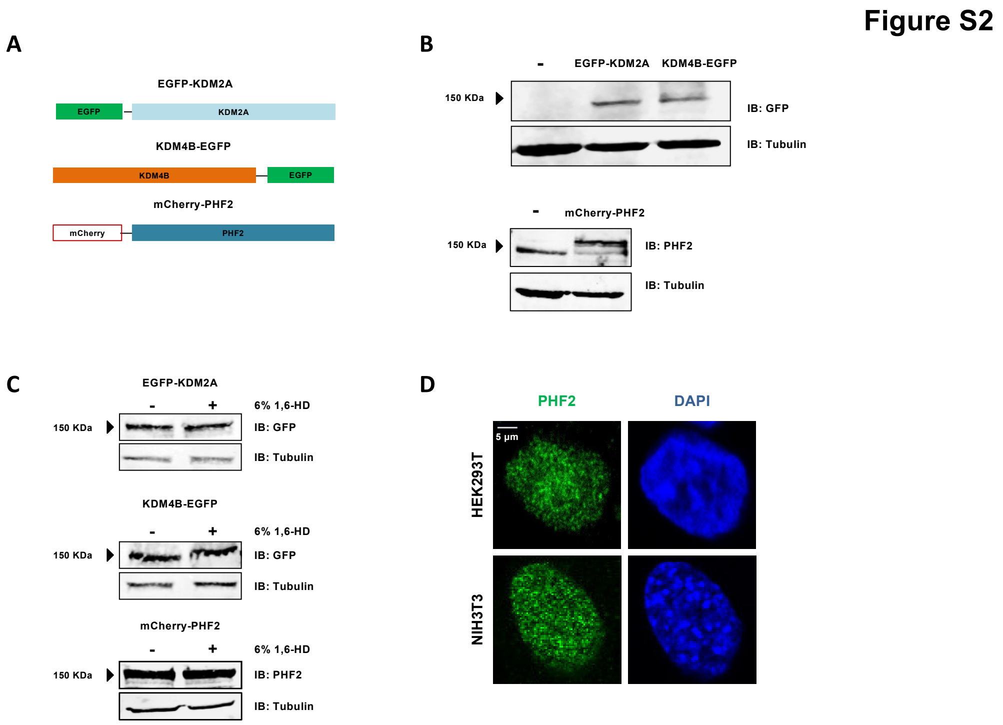

Figure S2. KDM2A, KDM4B and PHF2 form nuclear condensates. (A-B) EGFP-KDM2A, KDM4B-EGFP and mCherry-PHF2 expression vectors (A) were transfected into HEK293T cells. 24 h later total protein extracts were prepared and the expression levels of KDM2A, KDM4B and PHF2 were determined by immunoblot using anti GFP (KDM2A and KDM4B) and anti PHF2 (B). The image shown is representative of two independent experiments. (C) EGFP-KDM2A, KDM4B-EGFP and mCherry-PHF2 plasmids were transfected into HEK293T cells and treated or not with 6% 1,6-HD. Total protein extracts were prepared and the expression levels of KDM2A, KDM4B and PHF2 were determined by immunoblot using the indicated antibodies. The image shown is representative of two independent experiments. (D) HEK293T and NIH3T3 cells were fixed and endogenous PHF2 was visualized by immunostaining assay. Confocal microscopy images display puncta formed by endogenous PHF2. The images are representative of three biologically independent experiments. Scale bar, 5 µm.

Figure S3

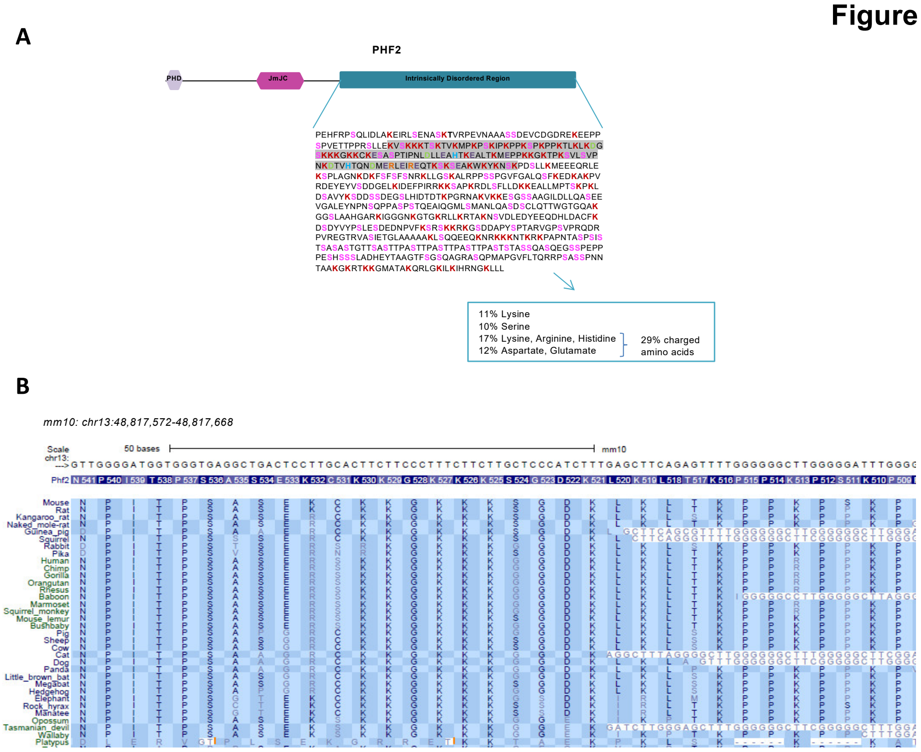

Figure S3. PHF2 IDR is rich in charged amino acids. (A) Schematic representation of the amino acid composition of PHF2 IDR. The percentages of lysines (purple), serines (pink), as well as basic, acid and charged amino acids are indicated at the bottom part of the panel. The grey-highlighted track corresponds to the lysine-enriched region (amino acids 487-806) deleted in PHF2 ΔCharged. (B) UCSC tracks displaying the conservation of PHF2 IDR charged region (541-509 AA) among vertebrate species using Multiz alignments (Blanchette et al., 2004).

References

Blanchette, M. *et al.* (2004). Aligning Multiple Genomic Sequences With the Threaded Blockset Aligner. *Genome Res.* **14,** 708–715.

Peng, K. *et al.* (2005). Optimizing long intrinsic disorder predictors with protein evolutionary information. *J. Bioinform. Comput. Biol.* **3,** 35–60.
